# Examination of age- and sex-related changes in protein expression within the hippocampus and prefrontal cortex during withdrawal from a subchronic history of binge-drinking in C57BL/6J mice

**DOI:** 10.3389/fnbeh.2025.1619889

**Published:** 2025-07-14

**Authors:** C. Leonardo Jimenez Chavez, Lauren E. Madory, Chris J. E. Denning, Edward C. Lee, Dylan T. Nguyen, Gavin P. Scheldrup, Karen K. Szumlinski

**Affiliations:** ^1^Department of Psychological and Brain Sciences, University of California Santa Barbara, Santa Barbara, CA, United States; ^2^Department of Molecular, Cellular and Developmental Biology, University of California Santa Barbara, Santa Barbara, CA, United States; ^3^Neuroscience Research Institute, University of California Santa Barbara, Santa Barbara, CA, United States

**Keywords:** adolescence, group 1 metabotropic glutamate receptors, tau, prefrontal cortex, hippocampus, sex differences

## Abstract

**Introduction:**

Early-onset binge-drinking and biological sex are critical risk factors for the development of cognitive decline and neurodegeneration associated with Alzheimer’s disease and related dementias (ADRDs). Recently, we demonstrated that a prior history of binge-drinking during adolescence induces what appears to be latent (>6 months post-drinking) changes in the expression of glutamate receptors and neuropathology markers within brain regions governing working and spatial memory, many of which precede the manifestation of overt cognitive anomalies.

**Methods:**

To determine whether alcohol-induced changes in protein expression manifest within the hippocampus and prefrontal cortex at earlier times post-drinking, we conducted immunoblotting on tissue from mice with a subchronic history of binge-drinking (14 days of 2-h access to 10, 20 and 40% ethanol) during either adolescence or adulthood.

**Results:**

We previously reported that this binge-drinking regimen produces mild, age- and sex-selective, changes in working memory and spatial recall when behavior was assayed starting a 1 or 30 days withdrawal. Here, we provide evidence a subchronic binge-drinking history is sufficient to alter the expression of certain glutamate receptors and ADRD-related proteins during the first few months following drinking cessation. Further, these alcohol-induced protein changes are regionally specific and sex-selective.

**Discussion:**

The present results add to our growing understanding of the long-term consequences of adolescent-onset binge-drinking of potential relevance to understanding individual variability in the cognitive consequences of heavy drinking.

## Introduction

A history of excessive alcohol-drinking is one of the most common risk factors for the development of dementia and cognitive decline (Wiegmann et al., 2020; Nunes et al., 2019; Schwarzinger et al., 2018) and there exists a high rate of co-occurrence between alcohol use disorder (AUD) and dementia, particularly Alzheimer’s Disease and related dementia (ADRD) (Hersi et al., 2017; Hoffman et al., 2019). The age of drinking-onset and amount of alcohol consumed during adolescence are major predictors of both AUD and early-onset ADRDs (Huang et al., 2018; Ledesma et al., 2021), with evidence from both rodent and human studies demonstrating that excessive alcohol intake during adolescence perturbs the development of the mesocorticolimbic system, which is presumed to contribute to cognitive anomalies in later life (Cservenka and Brumback, 2017; Novier et al., 2015). Consistent with this, we (Chavez et al., 2024; Jimenez Chavez et al., 2023; Szumlinski et al., 2019) and others (e.g., Van Hees et al., 2021) demonstrated recently that both subchronic (14-day) and more chronic (30-day) histories of binge-drinking during adolescence are sufficient to induce certain cognitive impairments in both the short (<2 months) and longer (≥ 6 months) term in C57BL/6J (B6J) mice. Further, the longer term (i.e., ≥ 6 months) cognitive effects of a prior adolescent binge-drinking history appear to be more apparent in female mice, while those observed in earlier withdrawal (≤ 30–40 days) appear to be less sex-dependent. Such findings align with evidence from rodent studies of forced “binge-like” alcohol exposure during adolescence (e.g., Pascual et al., 2007), as well as studies of humans indicating that women tend to exhibit higher vulnerability to alcohol-induced dementia in later life than men (Agabio et al., 2017; Ferretti et al., 2018; Hebert et al., 2013).

The onset of psychiatric symptoms indicative of, and comorbid with, an AUD has been linked to compensatory changes related to an overactive glutamate system that can persist during alcohol abstinence (e.g., Bergink et al., 2004; Hoffman and Tabakoff, 1994; Kamal et al., 2020; Koob, 2021), notably within key brain regions gating executive function, learning, and memory such as the prefrontal cortex (PFC) (Crews and Vetreno, 2014; Kalluri et al., 1998; Medina et al., 2008), entorhinal cortex (Ibáñez et al., 1995; Crews et al., 2000), and hippocampus (De Bellis et al., 2000; Kalluri et al., 1998; Nagel et al., 2005). Consistent with this, glutamate-dependent excitotoxicity has long been implicated in neurodegenerative diseases, as well as the capacity of alcohol to accelerate disease progression (e.g., Heymann et al., 2016; Kamal et al., 2020; Mira et al., 2019; Peng et al., 2020). Sex differences have been reported in the effects of chronic alcohol consumption on the expression of NMDA receptor subunits within the cortex and hippocampus (Chavez et al., 2024; Devaud and Morrow, 1999). Despite this, and the facts that women are approximately twice as likely to develop ADRD than men (Ferretti et al., 2018; Hebert et al., 2013) and the gender gap in binge-alcohol consumption is nearly closed (White, 2020), little preclinical research work has examined for sex differences in the effects of repeated alcohol exposure during adolescence on the expression of glutamate-related proteins and their relationship to indices of neuropathology within PFC and hippocampus. These regions are of particular interest as human studies indicate a more pronounced volumetric reduction within the PFC of women versus men with adolescent-onset heavy drinking (Medina et al., 2008), while hippocampal volumes appear to be similarly impacted by adolescent drinking in both sexes (De Bellis et al., 2000; Nagel et al., 2005).

Recently, we showed that female mice with a prior adolescent binge-drinking history exhibit more anomalies in the expression of glutamate receptor-related proteins, indices of kinase activity and markers of neuropathology within PFC, amygdala and entorhinal cortex when assayed following very prolonged periods of alcohol abstinence (6–12 months) (Chavez et al., 2024). These immunoblotting data indicate that a prior history of adolescent-onset binge-drinking produces latent or long-lasting (6–12 month) changes in brain biochemistry to which females may be more susceptible. Whether or not the same might be true with respect to sex differences in binge drinking-induced changes in brain biochemistry earlier during alcohol withdrawal remains to be determined. The present study sought to address this question by examining for sex by age interactions in the expression of glutamate receptor-related proteins, as well as protein indices of ADRD-related neuropathology within the hippocampus and PFC of the B6J mice from our prior behavioral study examining for sex differences in the effect of withdrawal from subchronic (14 days) binge-drinking during either adolescence or adulthood on negative affect and spatial cognition (Jimenez Chavez et al., 2023). Specifically, we immunoblotted for the obligatory GluN1 subunit of the NMDA receptor to index total NMDA receptor expression, as well as the alcohol-sensitive GluN2B NMDA receptor subunit (e.g., Wills et al., 2017). Both of these NMDA receptor subunits are up-regulated within hippocampus and PFC by alcohol exposure (e.g., Devaud and Morrow, 1999; Littleton et al., 2001; Mira et al., 2019; Nagy, 2004). Moreover, these subunits are up-regulated in adult rodents with a prior history of adolescent alcohol exposure (Carpenter-Hyland and Chandler, 2007; Swartzwelder et al., 2016; but see also Chavez et al., 2024). As the mGlu1 and mGlu5 subtypes of metabotropic glutamate receptors (**mGluRs**) are also upregulated in both adult mice with a history of adult-onset (Cozzoli et al., 2009, 2012, 2014; Szumlinski et al., 2023) or adolescent-onset binge-drinking (Chavez et al., 2024; Lee et al., 2016; Lee et al., 2017b), we examined their expression along with that of their highly alcohol-sensitive Homer scaffolding proteins (Campbell et al., 2019; Chavez et al., 2024; Cozzoli et al., 2009, 2012, 2014; Lee et al., 2016; Szumlinski et al., 2023). As in our recent reports (Campbell et al., 2019; Chavez et al., 2024), we also examined for p(Tyr204)-ERK1/2 expression to index cellular activity. Finally, we immunoblotted for certain proteins that currently serve as strong and reliable biomarkers of AD in human brain [e.g., amyloid precursor protein (APP), amyloid-*β* peptides (Aβ), hyper-phosphorylated tau proteins and beta secretase (BACE); Banning et al., 2021; Cheignon et al., 2018; Dodart et al., 2002; Hamley, 2012; Hersi et al., 2017; Perl, 2010]. These proteins accumulate in brain during normal aging in both humans and rodents (Arriagada et al., 1992; Haroutunian et al., 1998; O'Brien et al., 2009; Troncoso et al., 1998) and can be induced by prior alcohol experience in laboratory rodents (Hoffman et al., 2019; Liu et al., 2022; Salling et al., 2016; Szumlinski et al., 2023).

Based on studies of sex differences in alcohol-induced protein expression (Jimenez Chavez et al., 2022; Chavez et al., 2024), we hypothesized that females would exhibit more alcohol-related changes in protein expression than males, particularly in later withdrawal, when females exhibit more cognitive impairment than males (Jimenez Chavez et al., 2023).

## Materials and methods

### Subjects

**Figure 1:**
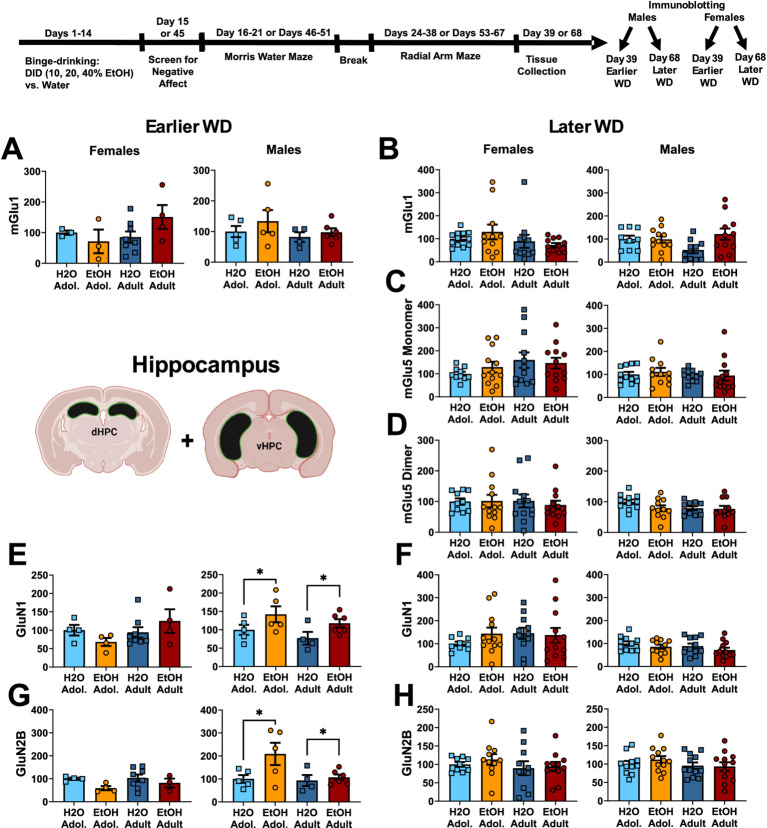
Summary of the changes in mGlu1 **(A,B)**, the monomer **(C)** and dimer form of mGlu5 **(D)**, GluN1 **(E,F)** and GluN2B **(G,H)** within the hippocampus of mice with a 2-week history of binge-drinking (EtOH) during either adolescence (Adol.) or adulthood (Adult) observed during early (left) or later alcohol withdrawal (right). H2O = water-drinking controls. Immunoblotting for mGlu5 was not conducted on hippocampus tissue from mice tested in early withdrawal. A cartoon illustrating the sites of tissue dissection is also presented. The data represent the means ± SEMs of the number of individual mice indicated. *p < 0.05 H2O vs. age-matched EtOH group (alcohol effect).

The male and female C57BL/6J (B6J) mice employed in the present study were the same as those described previously in Jimenez Chavez et al. (2023). In brief, adolescent and early adult mice were shipped from The Jackson Laboratory (Sacramento, CA, United States) at ages PND21 and PND 49, respectively. Mice were then housed under a reverse light cycle (lights off: 1,100 h) in same-sex and -age groups of 4 for 1 week prior to the commencement of study. Prior to tissue collection for the present study, subjects underwent a 14-day 2 h/day Drinking-in-the-Dark (DID) procedure, followed by a 1-day test battery for negative affect consisting of a light–dark box, a forced swim and a marble-burying assay, a 7-day Morris water maze and a 14-day radial arm maze procedure (see Figure 1). Procedural details of these behavioral assays are provided in Jimenez Chavez et al. (2023). All experimental procedures aligned with The Guide for the Care and Use of National Research Council (2011) and all protocols were approved by the Institutional Animal Care and Use Committee of the University of California, Santa Barbara.

### Immunoblotting

**Figure 2:**
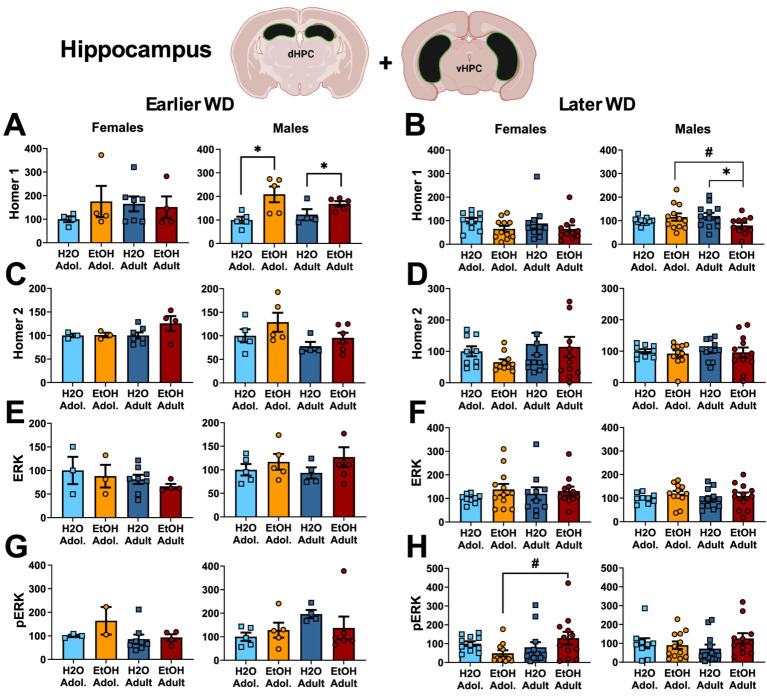
Summary of the changes in Homer1 **(A,B)**, Homer2 **(C,D)**, ERK **(E,F)** and p(Tyr204)-ERK **(G,H)** within the hippocampus of mice with a 2-week history of binge-drinking (EtOH) during either adolescence (Adol.) or adulthood (Adult) observed during early (left) or later alcohol withdrawal (right). H2O = water-drinking controls. A cartoon illustrating the sites of tissue dissection is also presented. The data represent the means ± SEMs of the number of individual mice indicated. *p < 0.05 H2O vs. age-matched EtOH group (alcohol effect); #*p* < 0.05 Adol. vs. Adult (age effect).

**Figure 3:**
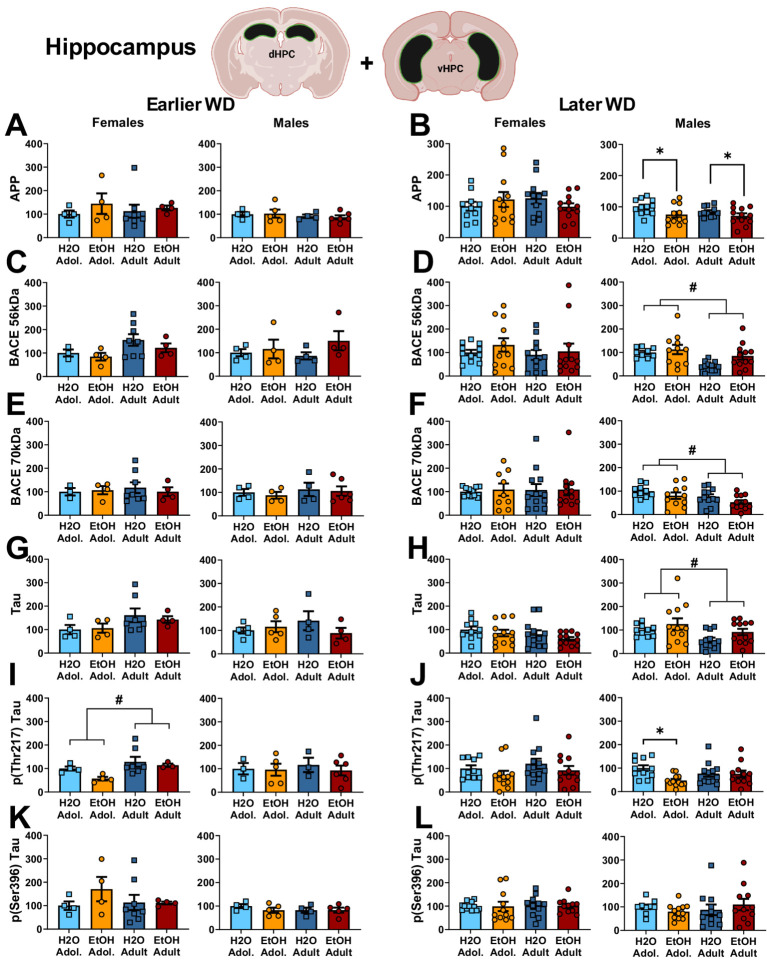
Summary of the changes in APP **(A,B)**, the 56 kDa **(C,D)** and the 70 kDa isoforms of BACE **(E,F)**, Tau **(G,H)**, p(Thr217)-Tau **(I,J)** and p(Ser396)-Tau **(K,L)** within the hippocampus of mice with a 2-week history of binge-drinking (EtOH) during either adolescence (Adol.) or adulthood (Adult) observed during early (left) or later alcohol withdrawal (right). H2O = water-drinking controls. A cartoon illustrating the sites of tissue dissection is also presented. The data represent the means ± SEMs of the number of individual mice indicated. *p < 0.05 H2O vs. age-matched EtOH group (alcohol effect); #*p* < 0.05 Adol. vs. Adult (age effect).

**Figure 4:**
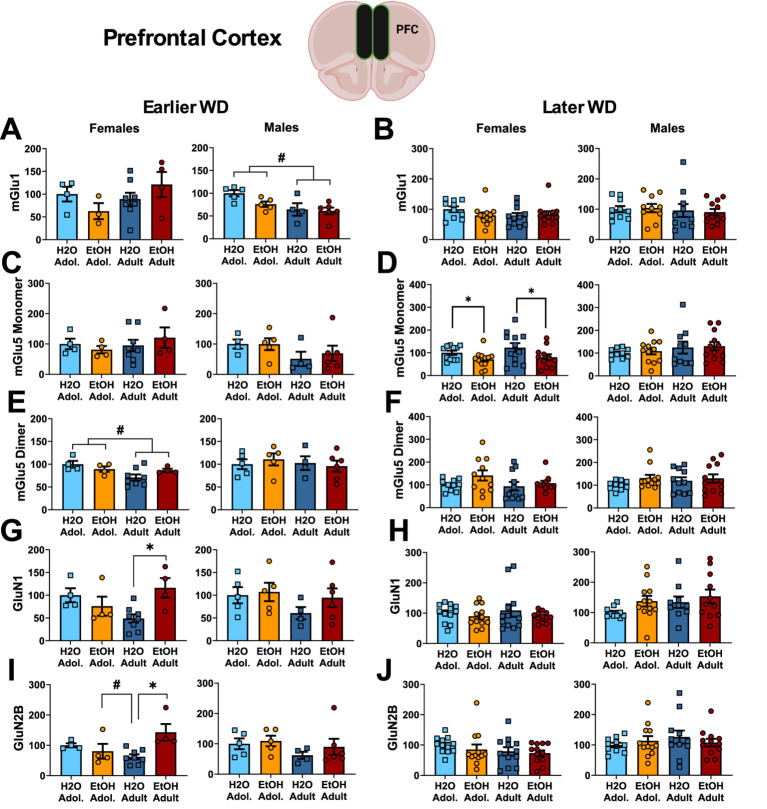
Summary of the changes in mGlu1 **(A,B)**, the monomer **(C,D)** and dimer form of mGlu5 **(E,F)**, GluN1 **(G,H)** and GluN2B **(I,J)** within the prefrontal cortex of mice with a 2-week history of binge-drinking (EtOH) during either adolescence (Adol.) or adulthood (Adult) observed during early (left) or later alcohol withdrawal (right). H2O = water-drinking controls. A cartoon illustrating the sites of tissue dissection is also presented. The data represent the means ± SEMs of the number of individual mice indicated. *p < 0.05 H2O vs. age-matched EtOH group (alcohol effect).

One day following the end of radial arm maze procedures (e.g., 25 or 54 days following the last drinking session for mice tested at the earlier versus later withdrawal time-point; see Figure 1), mice were decapitated, brains were extracted, cooled on ice, and then sectioned in 1 mm-thick coronal slices. Using blunt forceps, the entire PFC was dissected out and then both the ventral and dorsal hippocampus removed and hippocampal subregions combined into a single sample as illustrated in Figures 1–3 for hippocampus and Figures 2–4 for PFC.

Due to the large number of experimental groups, immunoblotting was conducted separately on the tissue from male and female mice, with separate experiments examining for protein expression at the different withdrawal time-points, akin to recent large-scale projects employing immunoblotting by our group (e.g., Chavez et al., 2024; Denning et al., 2024; Szumlinski et al., 2023). Gel electrophoresis was conducted on 16 gels total for each brain region, with each gel containing the tissue from 2 to 3 same-sex mice from each of our four conditions: adolescent water-drinking (H2O-Adol.), adolescent alcohol-drinking (EtOH-Adol.), adult water-drinking (H2O-Adult) and adult alcohol-drinking (EtOH-Adult). For each sex, 4 gels compared tissue from mice euthanized following testing in earlier withdrawal and 4 gels compared tissue from mice euthanized following testing in later withdrawal. The tissue homogenization and immunoblotting procedures employed in the present study were very similar to those detailed in our recent reports (e.g., Chavez et al., 2024; Denning et al., 2024; Huerta Sanchez et al., 2023; Szumlinski et al., 2023). We employed the same antibodies as those in Chavez et al. (2024), including the rabbit primary antibodies: mGlu5 (metabotropic glutamate receptor 5; 1:1,000 dilution; Millipore; AB5675), GluN1 (NMDA receptor subunit 1; 1:500 dilution; Cell Signaling Technology; 5704S), Homer2a/b (1:500 dilution; Synaptic Systems; 160,203), p-(Tyr204)ERK1/2 (1:750 dilution; R&D systems; AF1018), APP (1:1,000 dilution; Millipore-Sigma; 07–667), amyloid beta (1:500 dilution; Abcam, ab180956), p (Ser396)-tau (1:750 dilution; Abcam; ab109390) and p (Thr217)-tau (1:500 dilution; Invitrogen, 44–744) and the mouse primary antibodies: mGlu1 (metabotropic glutamate receptor 1; 1:500 dilution; BD Biosciences; 610,965), GluN2B (NMDA subunit 2B; 1:500 dilution; Invitrogen; MA1-2014), Homer1b/c (1:1,000 dilution; Santa Cruz Biotechnology, Santa Cruz, CA, USA; sc-25271), ERK1/2 (1:1,000 dilution; Invitrogen, MA5-15605), tau (1:750 dilution; Invitrogen, AHB0042) and BACE (1:500 dilution; Millipore Sigma; MAB5308). Note that as reported in our earlier studies (Chavez et al., 2024; Huerta Sanchez et al., 2023), our selected mGlu1 antibody does not reliably detect the dimer form of the receptor and thus, only the monomer form of mGlu1 is reported herein. To control for protein loading and transfer, we immunoblotted for calnexin expression using either a rabbit or mouse primary anti-calnexin antibody (for rabbit, 1:1000 dilution; Enzo Life Sciences; ADI-SPA-860; for mouse, 1:500 dilution; Invitrogen, MA5-31501).

Membranes were then washed with phosphate-buffered saline with tween, incubated in either a goat anti-rabbit IRDye 800CW secondary antibody (1:10,000 dilution; Li-Cor; 925–3,221) or a goat anti-mouse IRDye 680RD secondary antibody (1:10,000 dilution; Li-Cor; 925–68,070), and imaged on an Odyssey Infrared Imaging System (Li-Cor Biosciences, Lincoln, NE, USA). Raw values for each band were measured, and first normalized to their corresponding calnexin signal and then to the average value of the H2O-Adol. water controls for that particular gel (see more details below). It should be noted that several technical difficulties, including accidental tissue warming, were encountered when immunoblotting the tissue collected at the earlier withdrawal time-point and there was insufficient tissue to repeat immunoblotting procedures. As such, the sample sizes for all the proteins from the earlier withdrawal time-point are a small fraction of the original *n* = 12/sex/group originally collected and we were unsuccessful immunoblotting for mGlu5 expression in the hippocampus during early withdrawal. For transparency, the available data were plotted and analyzed but it is recognized that the statistical power of these analyses is low, likely precluding detection of group differences and subject factor interactions.

### Statistical analysis

As immunoblotting was conducted separately for male and female mice at the two different withdrawal time-points, the data for male and females were analyzed separately for protein expression determined at either the earlier or later withdrawal time-point (i.e., 25 or 54 days following the last binge-drinking session; see time-line in Figure 1) using Age x Drinking History ANOVAs. To increase statistical power to identify lower-level age differences in protein expression, alpha was set at 0.05 for all analyses and *post-hoc* LSD comparisons were performed. For all analyses where sphericity was violated, a Greenhouse–Geisser correction was used. Outliers were identified and excluded from the analyses using the ± 1.5 × IQR rule, however, in instances where too many outliers were identified, we adopted the ± 3 × IQR rule to ensure that only the most extreme outliers were removed. IBM SPSS Statistics software (version 27.0 for Macintosh) was used for all statistical tests, and GraphPad Prism software (version 9.3.1 for Macintosh) was used to create all graphs.

## Results

Table 1 summarizes the means ± SEMS of the average alcohol intake and the blood ethanol levels of the mice employed in this study. The results pertaining to the effects of binge-drinking on affective and cognitive behavioral measures are detailed in Jimenez Chavez et al. (2023).

**Table 1:** Summary of the average alcohol intake (g/kg) and blood alcohol levels (BACs; mg/dL) of the mice employed for immunoblotting in the present study.

Sex	Age	EtOH intake (g/kg)	BAC (mg/dL)
Males	Adol.	4.503 ± 0.163	97.186 ± 5.188
	Adult	3.411 ± 0.125	77.294 ± 4.031
Females	Adol.	5.167 ± 0.129	108.085 ± 4.563
	Adult	4.439 ± 0.122	96.503 ± 2.583

These data are depicted graphically and the results of the statistical analyses for these data can be found in Jimenez Chavez et al. (2023). Adol., adolescent-onset binge-drinking; Adult, adult-onset binge-drinking.

### Immunoblotting of hippocampal tissue

#### Glutamate receptor-related proteins

The results of the statistical analyses for glutamate receptor-related protein expression within the hippocampus are provided in Table 2 and representative immunoblots are provided in Supplementary Figure 1. When assayed on WD25, it appeared that mGlu1 monomer expression was higher in female mice with a history of adult-onset binge-drinking, compared to the other female groups. However, no significant group differences were detected for either female or male mice at the earlier time-point (Figure 1A) nor did we detect group differences in hippocampal mGlu1 monomer expression in mice assayed on WD54 (Figure 1B). Unfortunately, we could not immunoblot for the monomer and dimer form of mGlu5 on the hippocampal tissue collected from the earlier withdrawal time-point; thus, no data is available. However, we failed to detect any changes in the expression of the mGlu5 monomer (Figure 1C) or dimer (Figure 1D) within the hippocampus of mice on WD54. While no group differences were detected for hippocampal GluN1 expression in female mice at either withdrawal time-point (Figures 1E,F), GluN1 expression was higher in alcohol- versus water-drinking males when assayed on WD25 (Figure 1E). However, this alcohol effect was not apparent 1 month later (Figure 1F). Similarly, no group differences were detected for hippocampal GluN2B expression in female mice at either withdrawal time-point (Figure 1G, H). In contrast, GluN2B expression was higher in alcohol- versus water-drinking males in earlier (Figure 1G), but not later in withdrawal (Figure 1H).

**Table 2:** Summary of the statistical results from the study of glutamate-related protein expression and ERK activation within the hippocampus of male (M) and female (F) mice euthanized following behavioral testing in earlier (top) or later withdrawal (respectively, 25 and 54 days following the last binge-drinking session).

Protein of interest	Sex	Main effect of age	Main effect of drinking history	Age by drinking history interaction	Significant group comparisons
Earlier WD	Glutamate related proteins—hippocampus				
mGlu1	F	*p* = 0.290 η^2^*p* = 0.080	*p* = 0.535 η^2^*p* = 0.028	F(1,14) = 2.59, *p* = 0.130 η^2^*p* = 0.156	
	M	*p* = 0.253 η^2^p = 0.081	*p* = 0.294 η^2^*p* = 0.069	*F*(1,16) = 0.16, *p* = 0.697 η^2^p = 0.010	
GluN1	F	*p* = 0.207 η^2^p = 0.097	*p* = 0.976 η^2^p = 0.000	*F*(1,16) = 2.62, *p* = 0.125 η^2^p = 0.141	
	M	*p* = 0.164 η^2^*p* = 0.117	**F(1,16) = 6.59, *p* = 0.021** **η** ^**2**^ ***p* = 0.292**	F(1,16) = 0.00, *p* = 0.968 η^2^p = 0.000	**EtOH > H**_**2**_ **O** **(*p* = 0.021)**
GluN2B	F	*p* = 0.444 η^2^*p* = 0.040	*p* = 0.076 η^2^*p* = 0.195	F(1,15) = 0.35, *p* = 0.561 η^2^*p* = 0.023	
	M	*p* = 0.076 η^2^p = 0.183	**F(1,16) = 4.53, *p* = 0.049** **η** ^**2**^ **p = 0.221**	F(1,16) = 2.75, *p* = 0.117 η^2^p = 0.147	**EtOH > H**_**2**_ **O** **(*p* = 0.049)**
Homer 1b/c	F	*p* = 0.638 η^2^*p* = 0.015	*p* = 0.479 η^2^p = 0.034	F(1,15) = 1.10, *p* = 0.310 η^2^p = 0.068	
	M	*p* = 0.696 η^2^p = 0.010	**F(1,15) = 11.84, *p* = 0.004** **η** ^**2**^ **p = 0.441**	F(1,15) = 1.98, *p* = 0.180 η^2^p = 0.117	**EtOH > H**_**2**_ **O** **(*p* = 0.004)**
Homer 2a/b	F	*p* = 0.248 η^2^p = 0.101	*p* = 0.214 η^2^p = 0.116	*F*(1,13) = 1.47, *p* = 0.247 η^2^p = 0.101	
	M	*p* = 0.077 η^2^p = 0.183	*p* = 0.135 η^2^p = 0.134	F(1,16) = 0.14, *p* = 0.710 η^2^p = 0.009	
ERK	F	*p* = 0.233 η^2^p = 0.100	*p* = 0.419 η^2^*p* = 0.047	F(1,14) = 0.01, *p* = 0.933 η^2^p = 0.001	
	M	*p* = 0.926 η^2^p = 0.001	*p* = 0.165 η^2^p = 0.117	F(1,16) = 0.24, *p* = 0.633 η^2^p = 0.015	
pERK	F	*p* = 0.135 η^2^*p* = 0.163	*p* = 0.206 η^2^*p* = 0.120	F(1,13) = 1.14, *p* = 0.305 η^2^p = 0.081	
	M	p = 0.163 η^2^p = 0.118	*p* = 0.671 η^2^p = 0.012	F(1,16) = 1.46, *p* = 0.244 η^2^*p* = 0.084	
Later WD	Glutamate Related Proteins – Hippocampus				
mGlu1	F	*p* = 0.127 η^2^p = 0.056	*p* = 0.766 η^2^p = 0.002	*F*(1,41) = 1.11, *p* = 0.298 η^2^*p* = 0.026	
	M	*p* = 0.482 η^2^p = 0.013	*p* = 0.067 η^2^*p* = 0.086	*F*(1,38) = 3.93, *p* = 0.055 η^2^*p* = 0.094	
mGlu5 Dimer	F	*p* = 0.750 η^2^*p* = 0.002	*p* = 0.738 η^2^*p* = 0.003	*F*(1,42) = 0.18, *p* = 0.672 η^2^p = 0.004	
	M	*p* = 0.187 η^2^p = 0.044	*p* = 0.180 η^2^p = 0.046	F(1,39) = 1.24, *p* = 0.273 η^2^p = 0.031	
mGlu5 Monomer	F	*p* = 0.130 η^2^p = 0.054	*p* = 0.750 η^2^p = 0.002	F(1,42) = 0.72, *p* = 0.401 η^2^p = 0.017	
	M	*p* = 0.513 η^2^p = 0.010	*p* = 0.751 η^2^p = 0.002	*F*(1,42) = 0.15, *p* = 0.699 η^2^p = 0.004	
GluN1	F	*p* = 0.460 η^2^*p* = 0.014	*p* = 0.503 η^2^*p* = 0.011	*F*(1,40) = 0.98, *p* = 0.329 η^2^p = 0.024	
	M	*p* = 0.218 η^2^p = 0.037	*p* = 0.137 η^2^*p* = 0.053	F(1,41) = 0.02, *p* = 0.876 η^2^p = 0.001	
GluN2B	F	*p* = 0.308 η^2^p = 0.028	*p* = 0.521 η^2^p = 0.011	*F*(1,37) = 0.09, *p* = 0.764 η^2^p = 0.002	
	M	p = 0.247 η^2^p = 0.031	*p* = 0.615 η^2^p = 0.006	*F*(1,43) = 0.54, *p* = 0.468 η^2^p = 0.012	
Homer 1b/c	F	*p* = 0.700 η^2^p = 0.004	p = 0.078 η^2^*p* = 0.078	F(1,39) = 0.13, *p* = 0.724 η^2^p = 0.003	
	M	p = 0.513 η^2^p = 0.010	*p* = 0.336 η^2^p = 0.023	**F(1,41) = 4.71, *p* = 0.036 η** ^**2**^ ***p* = 0.103**	Adol: EtOH = H2O (*p* = 0.408) **Adult: EtOH < H2O** **(*p* = 0.030)**
Homer 2a/b	F	*p* = 0.204 η^2^*p* = 0.041	*p* = 0.440 η^2^p = 0.015	F(1,39) = 0.21, *p* = 0.653 η^2^*p* = 0.005	
	M	*p* = 0.769 η^2^p = 0.002	*p* = 0.522 η^2^p = 0.010	F(1,40) = 0.00, *p* = 0.974 η^2^p = 0.000	
ERK	F	*p* = 0.768 η^2^p = 0.002	*p* = 0.267 η^2^p = 0.032	*F*(1,38) = 0.39, *p* = 0.535 η^2^p = 0.010	
	M	*p* = 0.609 η^2^p = 0.006	*p* = 0.250 η^2^p = 0.032	F(1,41) = 0.07, *p* = 0.800 η^2^p = 0.002	
pERK	F	*p* = 0.223 η^2^p = 0.035	p = 0.953 η^2^p = 0.000	**F(1,42) = 4.11, p = 0.049 η** ^**2**^ **p = 0.089**	Adol.: EtOH = H2O (p = 0.156) Adult: EtOH = H2O (*p* = 0.162) **EtOH: Adol. < Adult (p = 0.026)** H2O: Adol. = Adult (*p* = 0.580)
	M	*p* = 0.875 η^2^p = 0.001	*p* = 0.361 η^2^p = 0.021	F(1,40) = 1.79, *p* = 0.188 η^2^p = 0.043	

Statistically significant results are bolded and highlighted.

#### Homer proteins and ERK activation

The results of the statistical analyses for Homer protein expression and ERK phosphorylation within the hippocampus are also provided in Table 2 and representative immunoblots are provided in Supplementary Figure 1. As illustrated in Figure 2, females exhibited no group differences in either Homer1b/c (Figures 2A,B) or Homer2a/b (Figures 2C,D) within hippocampus. In contrast, binge-drinking history increased Homer1b/c expression within the hippocampus of male mice on WD25 (Figure 2A) and a significant interaction was detected for this Homer isoform on WD54 (Figure 2B). This interaction reflected lower Homer1b/c expression in alcohol- versus water-experienced adults, with no alcohol-water difference detected in adolescent males (see Table 2). No changes in Homer2a/b were detected within the hippocampus of male mice during either withdrawal time-point (Figures 2C,D). Neither male nor female mice exhibited any change in hippocampal levels of ERK (Figures 2E,F) and males also did not differ in p(Tyr204)-ERK expression at either withdrawal time-point (Figures 2G,H). However, a significant interaction for this phospho-kinase was detected within the hippocampus of female mice on WD54 (Figures 2G,H). Post-hoc analyses indicated that this interaction did not reflect alcohol-water differences in either age group but rather reflected lower p(Tyr204)-ERK expression in alcohol-experienced adolescents versus adults, with no age difference noted in water-drinking controls (see Table 2).

#### Neuropathology markers

The results of the statistical analyses for the neuropathology markers examined within the hippocampus are provided in Table 3 and representative immunoblots are provided in Supplementary Figure 2. Neither male nor female mice exhibited changes in the hippocampal expression of APP on WD25 (Figure 3A), while binge-drinking males exhibited higher APP expression than their water controls ion WD54, irrespective of age of drinking-onset (Figure 3B). Females also did not exhibit any group differences in the expression of the 56 kDa (Figure C, D) or the 70 kDa (Figure E, F) BACE isoform at either withdrawal time-point. Males exhibited no group differences in the expression of either BACE isoform on WD25 (Figures 3C,E); however, the expression of both isoforms was higher in adolescent versus adult males and higher in alcohol- versus water-experienced males tested at the later withdrawal time-point (Figures 3D,F).

**Table 3:** Summary of the statistical results from the study of neuropathology-related proteins within the hippocampus of male (M) and female (F) mice euthanized following behavioral testing in earlier (top) or later (bottom) withdrawal (respectively, 25 and 54 days following the last binge-drinking session).

Protein of interest	Sex	Main effect of age	Main effect of drinking history	Age by drinking history interaction	Significant group comparisons
Earlier WD	Neuropathological protein expression—hippocampus				
Tau	F	*p* = 0.084 η^2^p = 0.186	*p* = 0.824 η^2^p = 0.003	F(1,15) = 0.22, *p* = 0.649 η^2^p = 0.014	
	M	*p* = 0.789 η^2^p = 0.005	*p* = 0.474 η^2^p = 0.037	F(1,14) = 1.82, *p* = 0.199 η^2^p = 0.115	
pThr(217) Tau	F	**F(1,14) = 5.11, *p* = 0.040** **η** ^**2**^ ***p* = 0.267**	*p* = 0.150 η^2^p = 0.142	*F*(1,14) = 0.57, *p* = 0.464 η^2^*p* = 0.039	**Adol. < Adult** **(*p* = 0.040)**
	M	*p* = 0.814 η^2^p = 0.004	*p* = 0.611 η^2^p = 0.020	F(1,13) = 0.13, *p* = 0.723 η^2^p = 0.010	
pSer(396) Tau	F	*p* = 0.539 η^2^p = 0.024	*p* = 0.357 η^2^p = 0.053	F(1,16) = 1.01, *p* = 0.329 η^2^p = 0.059	
	M	*p* = 0.439 η^2^p = 0.043	*p* = 0.431 η^2^*p* = 0.045	F(1,14) = 0.76, *p* = 0.398 η^2^p = 0.051	
BACE 56 kDa	F	*p* = 0.095, η^2^p = 0.174	*p* = 0.362 η^2^p = 0.056	F(1,15) = 0.12, *p* = 0.737 η^2^*p* = 0.008	
	M	*p* = 0.726, η^2^p = 0.011	*p* = 0.214 η^2^p = 0.126	*F*(1,12) = 0.64 *p* = 0.439 η^2^p = 0.051	
BACE 70 kDa	F	*p* = 0.833, η^2^p = 0.003	*p* = 0.832 η^2^p = 0.003	F(1,15) = 0.23, *p* = 0.637 η^2^p = 0.015	
	M	*p* = 0.479, η^2^p = 0.036	*p* = 0.647 η^2^p = 0.015	F(1,14) = 0.02, *p* = 0.902 η^2^p = 0.001	
APP	F	*p* = 0.931, η^2^p = 0.000	*p* = 0.361 η^2^p = 0.052	F(1,16) = 0.25, *p* = 0.627 η^2^p = 0.015	
	M	*p* = 0.331, η^2^p = 0.063	*p* = 0.932 η^2^p = 0.000	F(1,15) = 0.09, *p* = 0.767 η^2^p = 0.006	
Later WD	Neuropathological protein expression—hippocampus				
Tau	F	*p* = 0.105, η^2^p = 0.061	*p* = 0.200 η^2^p = 0.039	F(1,42) = 0.42, *p* = 0.836 η^2^p = 0.001	
	M	**F(1,42) = 5.77, *p* = 0.021, η** ^**2**^ **p = 0.121**	p = 0.069 η^2^*p* = 0.077	F(1,42) = 0.66, *p* = 0.799 η^2^p = 0.002	**Adol. > Adult** **(*p* = 0.021)**
pThr(217) Tau	F	*p* = 0.293, η^2^p = 0.026	*p* = 0.144 η^2^p = 0.050	F(1,42) = 0.00, *p* = 0.977 η^2^p = 0.000	
	M	*p* = 0.845, η^2^p = 0.001	**F(1,42) = 5.15, *p* = 0.028** **η** ^**2**^ **p = 0.109**	F**(1,42) = 4.45, *p* = 0.041 η**^**2**^**p = 0.096**	**Adol: EtOH < H2O** **(p = 0.003)** Adult: EtOH = H2O (*p* = 0.910)
pSer(396) Tau	F	*p* = 0.877, η^2^p = 0.001	*p* = 0.951 η^2^p = 0.000	*F*(1,41) = 0.00, *p* = 0.976 η^2^p = 0.000	
	M	*p* = 0.628, η^2^p = 0.006	*p* = 0.948 η^2^p = 0.000	F(1,39) = 1.38, *p* = 0.248 η^2^p = 0.034	
BACE 56 kDa	F	*p* = 0.466, η^2^p = 0.013	*p* = 0.370 η^2^p = 0.019	F(1,42) = 0.12, *p* = 0.734 η^2^p = 0.003	
	M	**F(1,40) = 9.94, *p* = 0.003, η** ^**2**^ **p = 0.199**	**F(1,40) = 4.22, *p* = 0.047, η** ^**2**^ ***p* = 0.095**	F(1,40) = 1.29, *p* = 0.262 η^2^p = 0.031	**Adol. > Adult** **(*p* = 0.003)** **EtOH > H**_**2**_ **O** **(p = 0.047)**
BACE 70 kDa	F	*p* = 0.876, η^2^p = 0.001	*p* = 0.808 η^2^p = 0.001	F(1,40) = 0.23, *p* = 0.880 η^2^p = 0.001	
	M	**F(1,41) = 6.03, *p* = 0.018 η** ^**2**^ **p = 0.128**	**F(1,41) = 4.27, *p* = 0.045, η** ^**2**^ **p = 0.094**	F(1,41) = 0.04, *p* = 0.835 η^2^p = 0.001	**Adol. > Adult** **(*p* = 0.018)** **EtOH < H**_**2**_ **O** **(*p* = 0.045)**
APP	F	*p* = 0.957, η^2^p = 0.000	*p* = 0.882 η^2^p = 0.001	F(1,42) = 1.90, *p* = 0.176 η^2^p = 0.043	
	M	*p* = 0.202, η^2^p = 0.039	**F(1,42) = 6.42, *p* = 0.015, η** ^**2**^ **p = 0.133**	F(1,42) = 0.59, *p* = 0.447 η^2^p = 0.014	**EtOH < H**_**2**_ **O** **(*p* = 0.015)**

Statistically significant results are bolded and highlighted.

Females also did not differ in terms of hippocampal levels of Tau at either withdrawal time-point (Figures 3G,H) nor were group differences detected in females for the expression of p(Ser396)-Tau (Figures 3K,L). Although p(Thr217)-Tau expression was higher in adult versus adolescent females on WD25 (Figure 3I), no group differences were noted later on WD54 (Figure 3J). In males, we detected no group differences in total Tau (Figures 3G,H), p(Ser217)-Tau (Figures 3I,J) or p(Thr396)-Tau (Figures 3K,L) at either time-point.

### Immunoblotting of prefrontal cortex tissue

#### Glutamate receptor-related proteins

The results of the statistical analyses for glutamate receptor-related protein expression within the PFC are provided in Table 4 and representative immunoblots are provided in Supplementary Figure 3. Female mice exhibited no group differences in the expression of the mGlu1 monomer within the PFC at either withdrawal time-point (Figures 4A,B). Although adolescent males exhibited higher mGlu1 monomer expression than male adults on WD25 (Figure 4A), no group differences were noted in later withdrawal (Figure 4B). mGlu5 monomer levels were unchanged in either female or male PFC on WD25 (Figure 4C), while binge-drinking females exhibited lower mGlu5 monomer levels on WD54, compared to their water-drinking controls (Figure 4D). Adult females also exhibited higher mGlu5 dimer expression within the PFC than adolescent females on WD25 (Figures 4E,F), but no differences in mGlu5 dimer expression were detected in the PFC of males. In females, Drinking History X Age interactions were detected for PFC expression of both the GluN1 (Figure 4G) and GluN2B (Figure 4I) subunit of the NMDA receptor on WD25. In both cases, these interactions reflected higher NMDA subunit expression in adult-onset binge-drinking females versus their water controls (see Table 4). These alcohol effects were not apparent in female mice tested later in withdrawal (Figures 4H,J) nor were group differences in the expression of either NMDA subunit apparent within the PFC of male mice (Figures 4G–J).

**Table 4:** Summary of the statistical results from the study of glutamate-related protein expression and ERK activation within the prefrontal cortex of male (M) and female (F) mice euthanized following behavioral testing in earlier (top) or later (bottom) withdrawal (respectively, 25 and 54 days following the last binge-drinking session).

Protein of interest	Sex	Main effect of age	Main effect of drinking history	Age by drinking history interaction	Significant group comparisons
WD1	Glutamate related proteins—prefrontal cortex				
mGlu1	F	*p* = 0.254 η^2^p = 0.086	*p* = 0.902 η^2^p = 0.001	F(1,15) = 3.02, *p* = 0.103 η^2^p = 0.168	
	M	**F(1,16) = 9.01, *p* = 0.008** **η** ^**2**^ **p = 0.360**	*p* = 0.124 η^2^p = 0.141	F(1,16) = 1.52, *p* = 0.236 η^2^p = 0.087	**Adol. > Adult (*p* = 0.008)**
mGlu5 Dimer	F	**F(1,16) = 5.06, *p* = 0.039** **η** ^**2**^ **p = 0.240**	*p* = 0.736 η^2^p = 0.007	F(1,16) = 3.53, *p* = 0.079 η^2^p = 0.181	**Adol. > Adult (*p* = 0.039)**
	M	*p* = 0.634 η^2^p 0.014	*p* = 0.879 η^2^p = 0.001	F(1,16) = 0.45, *p* = 0.513 η^2^p = 0.027	
mGlu5 Monomer	F	*p* = 0.464 η^2^p = 0.034	*p* = 0.882 η^2^p = 0.001	F(1,16) = 0.93, *p* = 0.349 η^2^p = 0.055	
	M	*p* = 0.103 η^2^p = 0.167	*p* = 0.702 η^2^p = 0.010	F(1,15) = 0.17, *p* = 0.690 η^2^p = 0.011	
GluN1	F	*p* = 0.734 η^2^p = 0.007	*p* = 0.192 η^2^*p* = 0.104	**F(1,16) = 8.32, *p* = 0.011 η** ^**2**^ ***p* = 0.342**	Adol.: EtOH = H_2_O (*p* = 0.329) **Adult: EtOH > H**_**2**_**O (*p* = 0.005)**
	M	*p* = 0.196 η^2^p = 0.102	*p* = 0.303 η^2^p = 0.066	F(1,16) = 0.48, *p* = 0.500 η^2^p = 0.029	
GluN2B	F	*p* = 0.469 η^2^p = 0.033	*p* = 0.086 η^2^p = 0.173	**F(1,16) = 9.02, *p* = 0.008** η^2^*p* **= 0.361**	Adol.: EtOH = H_2_O (*p* = 0.448) **Adult: EtOH > H**_**2**_**O (*p* = 0.002)**
	M	*p* = 0.195 η^2^p = 0.103	*p* = 0.397 η^2^p = 0.045	F(1,16) = 0.19, *p* = 0.672 η^2^p = 0.012	
Homer 1b/c	F	*p* = 0.148 η^2^*p* = 0.135	p = 0.765 η^2^p = 0.006	F(1,15) = 0.00, *p* = 0.954 η^2^p = 0.000	
	M	*p* = 0.660 η^2^p = 0.013	*p* = 0.547 η^2^p = 0.025	*F*(1,15) = 0.04, *p* = 0.850 η^2^p = 0.002	
Homer 2a/b	F	*p* = 0.292 η^2^p = 0.069	*p* = 0.602 η^2^p = 0.017	F(1,16) = 0.06, *p* = 0.817 η^2^p = 0.003	
	M	*p* = 0.502 η^2^p = 0.029	*p* = 0.700 η^2^p = 0.010	F(1,16) = 2.72, *p* = 0.119 η^2^p = 0.145	
ERK	F	*p* = 0.419 η^2^p = 0.044	*p* = 0.597 η^2^p = 0.019	F(1,15) = 0.16, *p* = 0.699 η^2^p = 0.010	
	M	*p* = 0.913 η^2^p = 0.001	*p* = 0.169 η^2^p = 0.015	F(1,16) = 1.90, *p* = 0.187 η^2^p = 0.106	
pERK	F	*p* = 0.104 η^2^p = 0.166	*p* = 0.280 η^2^p = 0.077	F(1,15) = 1.17, *p* = 0.297 η^2^p = 0.072	
	M	*p* = 0.976 η^2^p = 0.000	*p* = 0.675 η^2^p = 0.011	F(1,16) = 0.47, *p* = 0.502 η^2^p = 0.029	
Later WD	Glutamate Related Proteins – Prefrontal Cortex				
mGlu1	F	*p* = 0.340 η^2^p = 0.022	*p* = 0.461 η^2^p = 0.013	F(1,41) = 1.86, p = 0.180 η^2^p = 0.043	
	M	*p* = 0.537 η^2^p = 0.010	*p* = 0.955 η^2^p = 0.000	F(1,38) = 0.10, *p* = 0.752 η^2^p = 0.003	
mGlu5 Dimer	F	*p* = 0.226 η^2^p = 0.037	*p* = 0.105 η^2^p = 0.066	F(1,39) = 0.72, *p* = 0.403 η^2^p = 0.018	
	M	*p* = 0.510 η^2^p = 0.010	*p* = 0.355 η^2^p = 0.020	F(1,42) = 0.01, *p* = 0.913 η^2^p = 0.000	
mGlu5 Monomer	F	*p* = 0.268 η^2^p = 0.029	**F(1,42) = 6.60, *p* = 0.014** **η** ^**2**^ **p = 0.136**	F(1,42) = 0.24, *p* = 0.627 η^2^p = 0.006	**EtOH < H**_**2**_ **O** **(*p* = 0.014)**
	M	*p* = 0.205 η^2^p = 0.040	*p* = 0.689 η^2^p = 0.004	F(1,40) = 0.01, *p* = 0.944 η^2^p = 0.000	
GluN1	F	*p* = 0.770 η^2^p = 0.002	*p* = 0.219 η^2^p = 0.034	*F*(1,44) = 0.17, *p* = 0.684 η^2^p = 0.004	
	M	*p* = 0.170 η^2^p = 0.048	*p* = 0.120 η^2^p = 0.061	F(1,39) = 0.27, *p* = 0.608 η^2^p = 0.007	
GluN2B	F	*p* = 0.185 η^2^p = 0.040	*p* = 0.411 η^2^p = 0.015	F(1,44) = 0.13, *p* = 0.716 η^2^p = 0.003	
	M	*p* = 0.515 η^2^p = 0.010	*p* = 0.872 η^2^p = 0.001	F(1,41) = 1.07, *p* = 0.307 η^2^p = 0.025	
Homer 1b/c	F	*p* = 0.301 η^2^p = 0.024	*p* = 0.983 η^2^p = 0.000	F(1,44) = 0.76, *p* = 0.387 η^2^p = 0.017	
	M	*p* = 0.590 η^2^p = 0.007	*p* = 0.993 η^2^p = 0.000	F(1,42) = 0.47, *p* = 0.496 η^2^p = 0.011	
Homer 2a/b	F	*p* = 0.479 η^2^p = 0.013	*p* = 0.351 η^2^p = 0.022	F(1,40) = 0.79, *p* = 0.381 η^2^p = 0.019	
	M	*p* = 0.085 η^2^p = 0.069	*p* = 0.475 η^2^p = 0.012	F(1,42) = 3.10, *p* = 0.086 η^2^p = 0.069	
ERK	F	*p* = 0.533 η^2^p = 0.009	*p* = 0.881 η^2^p = 0.001	F(1,44) = 0.92, *p* = 0.342 η^2^p = 0.021	
	M	*p* = 0.833 η^2^p = 0.001	*p* = 0.435 η^2^p = 0.015	F(1,41) = 0.70, *p* = 0.407 η^2^p = 0.017	
pERK	F	*p* = 0.278 η^2^p = 0.031	*p* = 0.381 η^2^p = 0.020	**F(1,40) = 9.42, *p* = 0.004 η** ^**2**^ **p = 0.199**	**Adol.: EtOH > H2O** **(*p* = 0.011)** Adult: EtOH = H2O (*p* = 0.113)
	M	*p* = 0.606 η^2^p = 0.007	*p* = 0.569 η^2^p = 0.008	F(1,40) = 0.42, *p* = 0.521 η^2^p = 0.010	

Statistically significant results are bolded and highlighted.

#### Homer proteins and ERK activation

**Figure 5:**
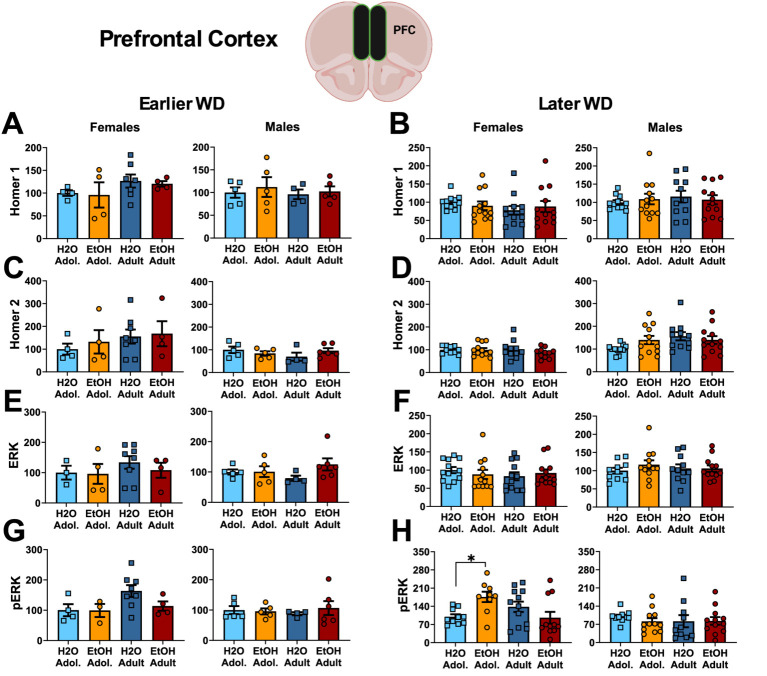
Summary of the changes in Homer1 **(A,B)**, Homer2 **(C,D)**, ERK **(E,F)** and p(Tyr204)-ERK **(G,H)** within the prefrontal cortex of mice with a 2-week history of binge-drinking (EtOH) during either adolescence (Adol.) or adulthood (Adult) observed during early (left) or later alcohol withdrawal (right). H2O = water-drinking controls. A cartoon illustrating the sites of tissue dissection is also presented. The data represent the means ± SEMs of the number of individual mice indicated. *p < 0.05 H2O vs. age-matched EtOH group (alcohol effect).

The results of the statistical analyses for Homer protein expression and ERK phosphorylation within the PFC are also provided in Table 4 and representative immunoblots are provided in Supplementary Figure 3. We detected no group differences in the PFC expression of either Homer1b/c (Figures 5A,B) or Homer2a/b (Figures 5C,D). We also did not detect any group differences in total ERK expression within PFC (Figures 5E,F). While mice of neither sex exhibited changes in p(Tyr204)-ERK expression within the PFC on WD25 (Figure 5G), a significant Drinking History X Age interaction was detected in female mice later in withdrawal (Figure 5H), that reflected higher p(Tyr204)-ERK expression in adolescent-onset binge-drinking mice, compared to their adult counterparts, with no age difference noted for water-drinking controls (see Table 4).

**Figure 6:**
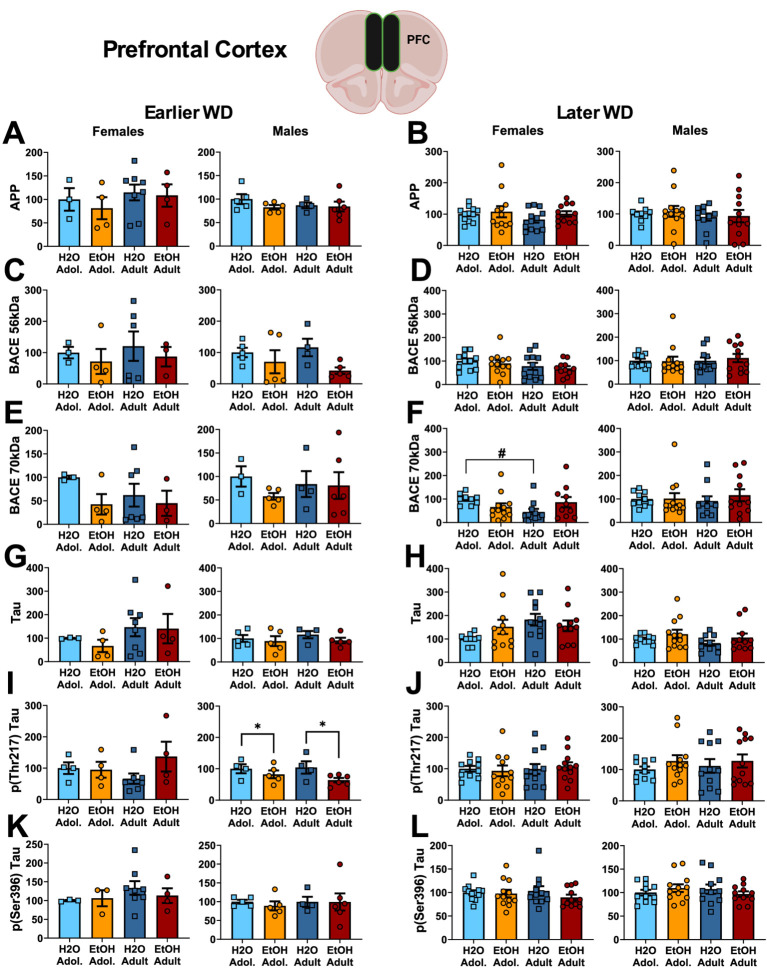
Summary of the changes in APP **(A,B)**, the 56 kDa **(C,D)** and the 70 kDa isoforms of BACE **(E,F)**, Tau **(G,H)**, p(Thr217)-Tau **(I,J)** and p(Ser396)-Tau **(K,L)** within the prefrontal cortex of mice with a 2-week history of binge-drinking (EtOH) during either adolescence (Adol.) or adulthood (Adult) observed during early (left) or later alcohol withdrawal (right). H2O = water-drinking controls. A cartoon illustrating the sites of tissue dissection is also presented. The data represent the means ± SEMs of the number of individual mice indicated. *p < 0.05 H2O vs. age-matched EtOH group (alcohol effect); #*p* < 0.05 Adol. vs. Adult (age effect).

*Neuropathology markers*. The results of the statistical analyses for the neuropathology markers examined within the hippocampus are provided in Table 5 and representative immunoblots are provided in Supplementary Figure 4. We detected no group differences in the PFC expression of APP (Figures 6A,B) or the 56 kDa BACE isoform within PFC (Figures 6C,D). While no group differences in the 70 kDa BACE isoform were detected on WD25 (Figure 6E), a Drinking History X Age interaction was detected in female mice later in withdrawal (Figure 6F). As presented in Table 5, this interaction did not reflect alcohol-water differences in either age group, but rather higher BACE 70 kDa expression in adolescent- versus adult-onset water-drinking controls, with no age difference detected in alcohol-drinking females.

**Table 5:** Summary of the statistical results from the study of neuropathology-related proteins within the prefrontal cortex of male (M) and female (F) mice euthanized following behavioral testing in earlier (top) or later (bottom) withdrawal (respectively, 25 and 54 days following the last binge-drinking session).

Protein of interest	Sex	Main effect of age	Main effect of drinking history	Age by drinking history interaction	Significant group comparisons
Early WD	Neuropathological protein expression—prefrontal cortex				
Tau	F	*p* = 0.220 η^2^p = 0.099	*p* = 0.681 η^2^p = 0.012	F(1,15) = 0.80, *p* = 0.781 η^2^p = 0.005	
	M	*p* = 0.568 η^2^p = 0.022	*p* = 0.286 η^2^p = 0.075	F(1,15) = 0.17, *p* = 0.686 η^2^p = 0.011	
pThr(217) Tau	F	*p* = 0.880 η^2^p = 0.002	*p* = 0.251 η^2^p = 0.087	F(1,15) = 1.90, p = 0.188 η^2^p = 0.113	
	M	*p* = 0.589 η^2^p = 0.020	**F(1,15) = 5.01, *p* = 0.041** **η** ^**2**^ **p = 0.250**	F(1,15) = 0.75, *p* = 0.399 η^2^p = 0.048	**EtOH < H**_**2**_ **O** **(*p* = 0.041)**
pSer(396) Tau	F	p = 0.370 η^2^p = 0.058	p = 0.751 η^2^p = 0.007	F(1,14) = 0.38, *p* = 0.550 η^2^p = 0.026	
	M	p = 0.764 η^2^p = 0.006	*p* = 0.735 η^2^p = 0.007	F(1,16) = 0.11, *p* = 0.740 η^2^p = 0.007	
BACE 56 kDa	F	*p* = 0.703 η^2^p = 0.013	*p* = 0.512 η^2^p = 0.037	F(1,12) = 0.00, *p* = 0.953 η^2^p = 0.000	
	M	*p* = 0.809 η^2^p = 0.004	*p* = 0.053 η^2^p = 0.228	F(1,15) = 0.81, *p* = 0.383 η^2^p = 0.051	
BACE 70 kDa	F	p = 0.512 η^2^p = 0.034	*p* = 0.184 η^2^p = 0.131	F(1,13) = 0.57, p = 0.464 η^2^p = 0.042	
	M	*p* = 0.893 η^2^p = 0.001	*p* = 0.380 η^2^p = 0.055	F(1,14) = 0.62, *p* = 0.446 η^2^p = 0.042	
APP	F	*p* = 0.375 η^2^p = 0.053	*p* = 0.591 η^2^p = 0.028	F(1,15) = 0.07, *p* = 0.790 η^2^p = 0.005	
	M	*p* = 0.527 η^2^p = 0.025	*p* = 0.289 η^2^p = 0.070	F(1,16) = 0.67, *p* = 0.426 η^2^p = 0.040	
Later WD	Neuropathological protein expression—prefrontal cortex				
Tau	F	*p* = 0.080 η^2^p = 0.079	*p* = 0.595 η^2^p = 0.008	F(1,38) = 2.61, *p* = 0.114 η^2^p = 0.064	
	M	*p* = 0.288 η^2^p = 0.029	*p* = 0.146 η^2^p = 0.054	*F*(1,39) = 0.01, *p* = 0.944 η^2^p = 0.000	
pThr(217) Tau	F	*p* = 0.627 η^2^p = 0.006	*p* = 0.955 η^2^p = 0.000	F(1,41) = 0.28, *p* = 0.599 η^2^p = 0.007	
	M	*p* = 0.755 η^2^p = 0.002	*p* = 0.263 η^2^p = 0.030	F(1,41) = 0.09, *p* = 0.765 η^2^p = 0.002	
pSer(396) Tau	F	*p* = 0.734 η^2^p = 0.003	*p* = 0.284 η^2^p = 0.027	F(1,42) = 0.63, *p* = 0.433 η^2^p = 0.015	
	M	*p* = 0.736 η^2^p = 0.003	*p* = 0.954 η^2^p = 0.000	F(1,41) = 1.68, *p* = 0.202 η^2^p = 0.039	
BACE 56 kDa	F	*p* = 0.094 η^2^p = 0.066	*p* = 0.455 η^2^p = 0.013	F(1,42) = 0.00, *p* = 0.964 η^2^p = 0.000	
	M	*p* = 0.696 η^2^p = 0.004	*p* = 0.759 η^2^p = 0.002	F(1,42) = 0.20, *p* = 0.654 η^2^p = 0.005	
BACE 70 kDa	F	*p* = 0.295 η^2^p = 0.029	*p* = 0.834 η^2^p = 0.001	**F(1,38) = 5.63, *p* = 0.023** **η** ^**2**^ **p = 0.129**	Adol.: EtOH = H2O (*p* = 0.074) Adult: EtOH = H2O (*p* = 0.137) EtOH: Adol. = Adult (*p* = 0.348) **H2O: Adol. > Adult** **(*p* = 0.023)**
	M	*p* = 0.894 η^2^p = 0.000	*p* = 0.543 η^2^p = 0.009	F(1,40) = 0.30, *p* = 0.587 η^2^p = 0.007	
APP	F	*p* = 0.296 η^2^p = 0.025	*p* = 0.267 η^2^p = 0.028	F(1,44) = 0.22, *p* = 0.643 η^2^p = 0.005	
	M	*p* = 0.439 η^2^p = 0.015	*p* = 0.721 η^2^p = 0.003	F(1,40) = 0.03, *p* = 0.872 η^2^p = 0.001	

Statistically significant results are bolded and highlighted.

No group differences were detected in the PFC expression of Tau (Figures 6G,H) or p(Ser396)-Tau (Figures 6K,L). Further, no group differences were detected for the PFC expression of p(Thr217)-Tau in female mice (Figures 6I,J). However, binge-drinking males exhibited higher p(Thr217)-Tau levels on WD25 only (Figures 6I,J).

## Discussion

### Summary of results

Consistent with the mild behavioral effects observed in our prior study (Jimenez Chavez et al., 2023), a subchronic (2-week) history of binge-drinking during either adolescence or adulthood elicited relatively few changes in the expression of glutamate receptors, neuropathology markers or ERK activation within the hippocampus and PFC when assayed at approximately 1 or 2 months following the last binge-drinking session. Although the loss of tissue samples and the resulting low sample sizes for the mice tested at the WD25 time-point likely contributed to the negative outcomes, we nevertheless detected an alcohol-dependent increase in the expression of the NMDA receptor subunits GluN1 and GluN2b, as well as their scaffolding protein, Homer1b/c, within the hippocampus of both adolescent- and adult-onset alcohol-drinking male mice approximately 1 month into withdrawal (WD25). Interestingly, when examined a month later (WD54), hippocampal Homer1b/c expression was significantly *lower* in adult-onset alcohol-drinking males, relative to their water-drinking controls, but the changes in NMDA subunit expression were no longer apparent. Finally, although age-related differences in BACE isoforms and Tau were detected in the hippocampus of male mice on WD54, the only alcohol-related effect was lower p(Thr217)-Tau expression in adolescent-onset binge-drinking males. In contrast to males, females exhibited no detectable alcohol-related protein changes within hippocampus at either withdrawal time-point examined herein.

In PFC, male mice exhibited no alcohol-related changes in glutamate receptor or Homer protein expression at either withdrawal time-point, nor did prior alcohol history affect ERK activation. The only alcohol-related effect observed within the PFC of male mice was an age-independent reduction in p(Thr217)-Tau expression on WD25 that was no longer apparent a month later. In contrast to males, a few alcohol-related changes were detected within the PFC of female mice that varied with the age of binge-drinking onset. These included increased GluN1 and GluN2b expression on WD25 in adult-onset binge-drinking females (with comparable trends noted also for the monomer forms of mGlu1 and mGlu5 monomer) and increased p(Tyr204)-ERK expression on WD54 in adolescent-onset binge-drinking females on WD54. No alcohol-related changes in neuropathology-related markers were apparent in the PFC of female mice. Taken together, the present results align with our prior work examining how subject factors, such as sex and age of binge-drinking onset (Chavez et al., 2024; Jimenez Chavez et al., 2023) interact in a complex manner with the duration of alcohol withdrawal to influence protein expression within PFC and hippocampus, even during the first few months post-drinking.

### Sex differences in alcohol-related changes in glutamate receptors at 1–2 months into withdrawal

This study is the first in our laboratory to compare subchronic (2-week) histories of adolescent- versus adult-onset binge-drinking on hippocampal and PFC protein expression in male versus female mice at any time-point following cessation of binge-drinking. While considerable evidence indicates that a history of adolescent alcohol exposure can induce long-lasting perturbations in indices of glutamate signaling within hippocampus (e.g., Beaudet et al., 2016; Contreras et al., 2019; Nwachukwu et al., 2022) and PFC (Galaj et al., 2020; Hardy et al., 1999; Salling et al., 2016; but see Raeder et al., 2008), the majority of these studies were conducted in male rodents only. However, a prior study of rats consuming a 6% alcohol liquid diet over a 14-day period demonstrated some clear sex differences in the effects of alcohol on NMDA receptor subunit expression within hippocampus and PFC (Devaud and Morrow, 1999). Consistent, in part, with evidence for some sex-selective effects of alcohol (Devaud and Morrow, 1999), alcohol binge-related changes in glutamate-related protein expression were detected on WD25 in the hippocampus of male mice only (Table 2). Given that the female rodents consumed more alcohol and achieved higher BACs than males in the current study (Table 1; Jimenez Chavez et al., 2022) and that by Devaud and Morrow (1999), the male-selectivity of the alcohol effects in hippocampus cannot be readily explained by sex differences in the amount of alcohol consumed or alcohol metabolism. It is possible that our low sample size for the WD25 time-point may have reduced our chances of detecting alcohol-water differences in female hippocampus. However, some female-selective changes in NMDA receptor subunits were detected in the PFC on WD25 (Table 4), despite the low sample size. Further, only females exhibited increased mGlu5 expression on WD54, when the sample size was sufficiently powered to detect alcohol effects in mice of both sexes. Yet, no alcohol-related changes in glutamate receptor proteins were detected in the PFC of males at either withdrawal time-point (Table 4). Such a double-dissociation in findings argues more in favor of genuine sex by brain region interactions in the alcohol sensitivity of glutamate receptor-related protein expression, rather than issues related to statistical power as accounting for our results, at least when protein expression is examined at 1–2 months into withdrawal.

Alternatively, a 2-week period of binge-drinking may simply be insufficient to alter glutamate receptor expression in the hippocampus of females or in the PFC of males. Indeed, comparable binge alcohol-related increases in the expression of both AMPA and NMDA receptor subunits, as well as Group 1 mGluRs, were observed in both mature adult and aged male and female subjects at 25 days withdrawal from a month-long multi-bottle DID binge-drinking procedure (Szumlinski et al., 2023). Moreover, patch-clamp electrophysiology studies indicate higher and lower intrinsic excitability of PFC neurons, respectively, in adult- and adolescent-onset alcohol-drinking male rats when assessed at 21 days withdrawal following a chronic, intermittent, alcohol procedure (Galaj et al., 2020). This being said, the duration of withdrawal is also a key modifier of alcohol’s effects on glutamate transmission within PFC as a female-selective reduction in NMDA and AMPA receptor cell surface expression and spontaneous excitatory postsynaptic potentials was detected within the prelimbic cortex of the PFC when assayed immediately following the last drinking session of a 4-week DID protocol when alcohol was still on board (Crowley et al., 2019), whereas increased intrinsic excitability of pyramidal neurons is observed in both male and female mice at 24 h withdrawal (Dao et al., 2021) and this increased excitability persists in both sexes for at least 6 months into withdrawal (Smith et al., 2025).

However, in contrast to the PFC, only males exhibited age- and alcohol-related changes in glutamate receptor expression within hippocampus in our prior study of older mice (Szumlinski et al., 2023). As we have yet to examine for sex differences in hippocampal protein expression during acute (1 day) alcohol withdrawal, we do not yet know if our results to date (Szumlinski et al., 2023; Table 2) reflect an insensitivity of the female hippocampus to alcohol drinking-induced changes in glutamate-related protein expression or sex differences in the persistence of any alcohol-induced changes that might occur earlier than 25 days into withdrawal. Arguing in favor of the latter possibility, both male and female alcohol-dependent adult rats exhibit increased hippocampal expression of GluN1 in acute alcohol withdrawal (Devaud and Morrow, 1999). To the best of our knowledge, only one other study has examined for sex differences in the effects of more protracted withdrawal (11 days) from a binge-like drinking paradigm (schedule-induced drinking) on hippocampal NMDA subunit mRNA expression and observed no alcohol-induced changes in mRNA in either sex at this time-point, despite a male-selective alcohol-induced impairment in spatial memory, as well as synaptic plasticity within hippocampus (Sanz-Martos et al., 2023). Given these disparate results, the sex differences in glutamate receptor-related protein expression observed herein at 1–2 months into alcohol withdrawal, and clinical evidence that women are more vulnerable to alcohol-induced cognitive impairment than men (e.g., Bahorik et al., 2021; Mumenthaler et al., 1999; Squeglia et al., 2009, 2011a, 2011b), it will be important in future work to systemically examine how the age of binge-drinking onset interacts with sex to impact both pre- and postsynaptic aspects of glutamate transmission within the PFC and hippocampus earlier during alcohol withdrawal in light of their important roles in executive and cognitive processing, respectively.

### Alcohol-related changes in neuropathology markers during protracted withdrawal in male and female mice

Despite detecting several age-related differences in the expression of proteins associated with ADRD-related neuropathology in the present study, particularly within hippocampus, we detected only a few alcohol-related changes in protein expression. Perhaps this result should not be surprising given the relatively young age of the mice at both binge-drinking onset and tissue collection (< 4 months of age). Supporting age at the time of study as a modifying factor in both alcohol-related and baseline neuropathology marker expression, p(Thr217)-Tau expression was *lower* within the PFC of binge-drinking males in early withdrawal, irrespective of their age of drinking-onset and age-related *decreases* in APP, the 56 and 70 kDa BACE isoforms and Tau were apparent within the hippocampus of male mice during later withdrawal, irrespective of prior alcohol history. Further, female water-drinking mice exhibited an age-related reduction in 70 kDa BACE expression within the PFC. These findings contrast with the results from our study of older male mice in which: (1) the expression of our neuropathology markers tended to be higher within hippocampus of 18- versus 6-month-old mice, with fewer age-related changed detected for PFC and (2) prior alcohol-drinking history either increased or did not affect the expression of our neuropathology markers within hippocampus and PFC (Szumlinski et al., 2023). As these latter data for alcohol-experienced older mice align with the limited literature on age- and alcohol-related changes in ADRD-related genes/proteins in wild-type mice (Hoffman et al., 2019; Liu et al., 2022; Salling et al., 2016; Wisniewski et al., 1973), the present results argue that the age at tissue collection may be important for not only detecting, but also the direction of, both baseline and alcohol-related changes in, ADRD-related marker expression.

Supporting this idea, adolescent-onset binge-drinking elicited alcohol-related increases in p(Thr217)-Tau expression in entorhinal cortex and PFC, BACE (70 kDa) in entorhinal cortex, APP in entorhinal cortex, PFC and amygdala, and Aβ within amygdala during very protracted withdrawal, while the expression of what is considered to be one of the earliest markers of ADRD, (Ser396)-Tau (Janelidze et al., 2020), is reduced in all three brain regions during very protracted withdrawal (Chavez et al., 2024). In fact, the only age-related difference in ADRD marker expression observed in the present study that is consistent with the extant literature was p(Thr217)-Tau within the hippocampus of females tested in early withdrawal. As p(Thr217)-Tau is considered a highly specific biomarker of AD in both preclinical and advanced stages of AD in humans (e.g., Fukumoto et al., 2002; Johnson and Stoothoff, 2004), it is tempting to speculate that female hippocampus may telescope through normal aging-related neuropathology. However, this age-related difference in p(Thr217)-Tau expression was no longer apparent in females at the later withdrawal time-point, suggesting that the early withdrawal effect may be a spurious result related to our low sample sizes. Given the ample evidence that heavy alcohol drinking increases dementia vulnerability (Huang et al., 2018; Mumenthaler et al., 1999; Sabia et al., 2018; Weyerer et al., 2011; Xu et al., 2017) and reduces the age of dementia-onset in both humans (Kilian et al., 2023; Koch et al., 2019; Zarezadeh et al., 2024) and laboratory rodents (Crews and Vetreno, 2014; Hoffman et al., 2019; Jimenez Chavez et al., 2020; Ledesma et al., 2021), it is clear that more work is required to gain a more thorough understanding of the biomolecular impact of developmental exposure to alcohol, not only during critical periods of neuroplasticity (e.g., adolescence), but also in later life, in order to better predict behavioral outcomes and the efficacy of therapeutic interventions.

## Conclusion

Herein we show that a 2-week history of binge-drinking by male and female, adult and adolescent, B6 mice is sufficient to elicit some sex- and region-selective changes in glutamate receptor-related and ADRD-related protein expression within the hippocampus and PFC that vary as a function of the duration of alcohol withdrawal and may contribute to mild cognitive impairment reported following adolescent- and adult-onset binge-drinking.

## Data Availability

The raw data supporting the conclusions of this article will be made available by the authors, without undue reservation.
