# Supplementary material for: Examination of age- and sex-related changes in protein expression within the hippocampus and prefrontal cortex during withdrawal from a subchronic history of binge-drinking in C57BL/6J mice
Source: Front Behav Neurosci. 2025 Jul 14;19:1619889. doi: 10.3389/fnbeh.2025.1619889 (PMC12301339; doi:10.3389/fnbeh.2025.1619889)
Supplement: Supplementary file 1 [file Data_Sheet_1.docx]

**SUPPLEMENTAL RESULTS**

**Supplemental Figure 1:**  Representative immunoblots for glutamate-related proteins and ERK activation within the hippocampus of male and female rats with a prior 2-week history of binge-drinking during either adolescence or adulthood. Immunoblotting was conducted during either early (WD25) or later withdrawal (WD54).

**Supplemental Figure 2:**  Representative immunoblots for neuropathology markers within the hippocampus of male and female rats with a prior 2-week history of binge-drinking during either adolescence or adulthood. Immunoblotting was conducted during either early (WD25) or later withdrawal (WD54).

**Supplemental Figure 3:**  Representative immunoblots for glutamate-related proteins and ERK activation within the PFC of male and female rats with a prior 2-week history of binge-drinking during either adolescence or adulthood. Immunoblotting was conducted during either early (WD25) or later withdrawal (WD54).

**Supplemental Figure 4:**  Representative immunoblots for neuropathology markers within the PFC of male and female rats with a prior 2-week history of binge-drinking during either adolescence or adulthood. Immunoblotting was conducted during either early (WD25) or later withdrawal (WD54).
